# Can We Avoid Radiation Exposure in Retrograde Intrarenal Surgery?

**DOI:** 10.3390/medicina62030428

**Published:** 2026-02-24

**Authors:** Süleyman Öner, Utku Bekyürek, Aydın Yenilmez

**Affiliations:** Department of Urology, Faculty of Medicine, Eskisehir Osmangazi University, 26040 Eskisehir, Turkey; utku.bekyurek@ogu.edu.tr (U.B.); ayenilmez@ogu.edu.tr (A.Y.)

**Keywords:** renal stone, urolithiasis treatment, RIRS, fluoroscopy-free RIRS, radiation

## Abstract

*Background and Objectives:* The use of fluoroscopy during retrograde intrarenal surgery (RIRS) results in cumulative ionizing radiation exposure to both the patient and the surgical team. We aimed to evaluate the efficacy and safety of fluoroscopy-free (FF) RIRS performed by experienced surgeons in the management of renal stones < 2 cm. *Materials and Methods:* A total of 255 patients who underwent RIRS for renal stones < 2 cm between 2023 and 2025 were retrospectively analyzed. Patients were randomly assigned to the groups. Fluoroscopy was used (FU) during RIRS in 123 patients, whereas fluoroscopy was not used during RIRS in 132 patients. All procedures were performed by a single experienced surgeon. For patients in both groups, the following variables were retrospectively reviewed: demographic characteristics, stone characteristics, localization, and diameter, operative time, fluoroscopy time and dose, postoperative complications, length of hospital stay, and stone-free rates (SFR). *Results:* The operative time was 34.7 ± 8.7 min in the FF group and 42.0 ± 12.9 min in the FU group, being significantly shorter in the FF group (*p* < 0.001). No fluoroscopy was used in the FF group, whereas in the FU group the fluoroscopy time and dose were recorded as 7.75 ± 3.6 s and 1.31 ± 0.61 mGy, respectively. There were no significant differences between the groups in terms of length of hospital stay or SFR. No intraoperative complications were observed in either group. Postoperative complications occurred in 29 (21.9%) patients in the FF group and 42 (34.1%) patients in the FU group; the difference between groups was statistically significant (*p* = 0.030). *Conclusions:* In appropriately selected patients with renal stones < 2 cm, fluoroscopy-free RIRS performed by experienced surgeons can be applied effectively and safely, with shorter operative times and lower complication rates.

## 1. Introduction

With the rising global prevalence of kidney stone disease, significant advances have been achieved in the field of endourology. Owing to technological developments, the instruments used in endoscopic procedures have become smaller, the use of flexible ureterorenoscopes has become widespread, and larger stones can now be safely fragmented with modern laser systems. In parallel with these developments and in line with patient preferences, Retrograde Intrarenal Surgery (RIRS) has been increasingly favored worldwide for the treatment of renal calculi [1]. With increasing surgical experience, it may be possible to avoid radiation during RIRS. More information is needed in the literature regarding flouroscopy-free (FF) RIRS techniques.

The routine use of fluoroscopy during RIRS is employed for several purposes, including delineation of the renal calyceal and ureteral anatomy via retrograde pyelography, confirmation of guidewire placement, assessment of ureteral access sheath positioning, facilitation of access to the stones, verification of stone-free status, and confirmation of stent placement [2]. Although the amount of radiation exposure from fluoroscopy during endourological interventions is lower than that associated with diagnostic imaging, it nonetheless contributes to cumulative whole-body radiation exposure. Consequently, the effects of radiation are cumulative [3].

With the increasing use of RIRS for the management of kidney stone disease, exposure of the patient, surgeon, and operating room staff to ionizing radiation from fluoroscopy has become an increasingly important concern. In addition to radiation exposure during fluoroscopy-guided procedures, surgeons and operating room staff also face an increased risk of morbidity related to the weight of lead aprons worn for protection. The recommended maximum occupational radiation dose limit is an average of <20 mSv/year over 5 years, or 50 mSv in any single year [4]. Due to the lack of consensus on an ideal imaging protocol for the follow-up of patients with urolithiasis, these individuals are frequently subjected to excessive computed tomography (CT) and Kidney-Ureter-Bladder (KUB) radiography. Consequently, this leads to an increased cumulative radiation exposure within this patient population [5]. Alarmingly, some studies have reported that 17.3–20% of patients with stone disease exceeded the critical annual radiation threshold of 50 mSv within the first year of follow-up [6,7]. These findings clearly indicate that the health risks associated with radiation exposure cannot be overlooked and should be strongly taken into account by treating urologists [8].

Numerous techniques have been described to reduce radiation exposure during endourological procedures. These approaches aim to minimize exposure by adhering to the “ALARA” (As low as reasonably achievable) principles. The core components of these protocols include enhancing education and awareness regarding radiation-related risks for both patients and healthcare personnel [9].

While preventing or reducing radiation exposure during endourological procedures, optimal surgical outcomes should be achieved without an increase in complications. Toward this end, Ngo et al. achieved a 24% reduction in intraoperative fluoroscopy use without a significant change in operative times [10]. Several studies have investigated the feasibility of FF RIRS in the treatment of urinary stone disease. Olgin et al. were the first to compare fluoroscopy-free endoscopic ureteral stone treatment with a control group using fluoroscopy; in their protocol, guide wire placement was achieved through tactile feedback and gentle maneuvers, and the authors reported comparable success and complication rates between the two groups [11]. Boeri et al. demonstrated that once surgical procedures are standardized and adequate training is provided, inexperienced surgeons can achieve reductions in radiation exposure comparable to those of experienced surgeons [12]. To contribute to the existing literature, in our study we aimed to investigate the effectiveness and safety of FF RIRS, performed by experienced hands, compared to fluoroscopy-used (FU) RIRS in the treatment of kidney stones < 2 cm, in terms of operation time, hospital stay, stone-free rate, and complications.

## 2. Materials and Methods

Ethical approval for this study was obtained from the Institutional Review Board of our University on 9 September 2025 (Approval No.: E-25403353-050.04-250180969).

Patients who underwent RIRS for the treatment of kidney stones smaller than 2 cm between January 2023 and April 2025 were examined. Patients under 18 years of age, pregnant women, those with congenital urogenital anomalies, those with ureteral stones, those with a history of ureteral stricture, and those with a history of radiotherapy were excluded because they would require a more specialized approach to the procedure. The remaining 255 patients were included in the study. In Operating Room 1, fluoroscopy use during RIRS was planned, whereas in Operating Room 2, RIRS was planned to be performed without fluoroscopy. Patients were allocated into two groups in a quasi-random manner according to their surgical appointment schedules: fluoroscopy was used during RIRS in the first group (FU), while no fluoroscopy was used in the other group (FF). All procedures were performed by a single surgeon experienced in RIRS (with >10 years of endourology experience and performing >150 RIRS procedures annually). For patients in both groups, the following variables were retrospectively reviewed: demographic characteristics, comorbid systemic diseases, ASA scores, history of anticoagulant use, laterality of the stone-bearing kidney, stone radiopacity, stone size, presence of multiple stones, intrarenal stone location, stone radiodensity, history of prior stone surgery, presence of a preoperative double-J stent, operative time, fluoroscopy time, fluoroscopy dose, postoperative complications, length of hospital stay, and stone-free rates (SFR). To minimize errors and reduce bias, the data collection process was conducted independently by two researchers using the Hospital Information Management System. The extracted data were then cross-checked by a third researcher, and statistical analysis was performed on the verified dataset. A complete-case analysis was employed to manage missing data; patients with incomplete medical records or lost to follow-up were excluded during the initial screening phase to prevent potential bias in result reporting.

Preoperatively, all patients underwent non-contrast CT. Stone size was calculated as the maximum diameter of the stone on CT in any axis. In cases with multiple renal stones, the cumulative stone burden was defined as the sum of the maximal diameters of each stone. Patients presenting with preoperative renal colic, significant hydronephrosis, or a history of pyelonephritis underwent placement of a preoperative double-J (DJ) stent or a nephrostomy tube. Before the procedure, urine cultures were sterilized in all patients, and a single-dose preoperative antibiotic prophylaxis was administered. In both groups, patients were operated on under general anesthesia in the lithotomy position. In the FU group, the urethra was entered with a 20-F endoscope (Karl Storz Corp., Tuttlingen, Germany); after bladder inspection, a guidewire was advanced into the kidney under fluoroscopic guidance and left in place. For ureteral dilation, a semi-rigid ureterorenoscope (6.5/7.5 F) (Richard Wolf Corp., Knittlingen, Germany) was advanced up to the ureteropelvic junction. Subsequently, a 7.5-F single-use flexible ureterorenoscope (Scivita Medical Technology Corp., Suzhou, China) was advanced into the kidney over the guidewire under fluoroscopic guidance. Renal stones were fragmented using a Ho:YAG laser (LISA Laser Products Corp., Katlenburg-Lindau, Germany). Laser settings varied between 0.8 and 1.2 J and 10 to 12 Hz, depending on stone density and/or surgeon preference. If the stone was radiopaque, stone-free status was assessed with the aid of fluoroscopy. Using a 20-F endoscope, the guidewire was re-advanced into the kidney under fluoroscopic guidance and left in place. A DJ stent (Plastimed Medical Products Corp., İstanbul, Turkey) was then inserted under fluoroscopic control. A portable C-arm fluoroscopy unit Ziehm Solo FD (Ziehm Imaging Corp., Nuremberg, Germany) was used in control mode at 42 kV, 2.5 mA, and 1 pulse/second. The device-reported air kerma per single shot was recorded as 0.17 mGy/second. The radiation dose generated by the device (mGy) was calculated by multiplying the fluoroscopy time (seconds) by the device-specific air kerma value. In the FF group, the urethra was entered with a 20-F endoscope; after bladder inspection, a curved-tip hydrophilic guidewire was advanced into the kidney, and appropriate positioning was presumed based on the tactile “coiling” sensation and the estimated length of wire inserted. A semi-rigid ureterorenoscopy (6.5/7.5 F) was then performed up to the ureteropelvic junction to confirm that the guidewire was correctly in place. Subsequently, a 7.5-F single-use flexible ureterorenoscope was advanced into the kidney over the guidewire under endoscopic visualization. Renal stones were fragmented using a Ho:YAG laser. Laser settings ranged from 0.8 to 1.2 J and 10 to 12 Hz, depending on stone density and/or surgeon preference. At the end of the procedure, all calyces were inspected to confirm stone-free status. Under direct visualization, the guidewire was re-advanced into the kidney and left in place. A DJ stent was then inserted using a 20-F endoscope, and the position of the distal stent coil was adjusted under direct endoscopic vision. In the FF group, a C-arm fluoroscopy unit was kept available in the operating room for emergencies; however, none of the cases required rescue fluoroscopy.

Patients were invited for follow-up 2–3 weeks after surgery. At follow-up, patients with radiopaque or semi-opaque stones underwent a plain kidney–ureter–bladder (KUB) radiograph, whereas those with non-opaque stones underwent ultrasonography; CT was performed when deemed necessary. Asymptomatic residual fragments ≤ 3 mm were considered stone-free for the purpose of calculating the stone-free rate (SFR). In patients considered stone-free, DJ stents were removed between postoperative day 14 (earliest) and day 21 (latest). Perioperative complications and complications occurring within 1 month after surgery were recorded.

All statistical analyses were performed using IBM SPSS Statistics v25 (IBM Corp., Armonk, NY, USA). The distributional properties of continuous variables were assessed using visual methods (histograms and Q–Q plots) and analytical tests. Continuous data not showing a normal distribution were expressed as medians. The Mann–Whitney U test was used to compare continuous variables between the FU and FF groups. Descriptive statistics were presented as mean ± standard deviation for continuous numerical variables. Categorical variables were presented as frequencies and percentages (%). The chi-square test was used to compare categorical variables between groups, and a *p* value < 0.05 was considered statistically significant. Due to the difference in age distribution identified during group randomization, a subgroup analysis was conducted. However, as the total sample size decreased following this analysis, further in-depth group comparisons were not pursued to avoid a potential reduction in statistical power.

## 3. Results

Of the 255 patients included in the study, 123 underwent surgery with fluoroscopy (FU) and 132 underwent surgery without fluoroscopy (FF). Regarding demographic and clinical characteristics, the mean age was 52.7 ± 14.2 years in the FF group and 55.5 ± 16.2 years in the FU group, with a statistically significant difference between groups (*p* = 0.046). The prevalence of hypertension was 37 (28%) in the FF group and 56 (45.5%) in the FU group, being significantly higher in the FU group (*p* = 0.004). There were no significant differences between the groups in terms of sex distribution, mean BMI, presence of diabetes mellitus (DM), ASA classification, anticoagulant use, prior stone surgery, or the presence of a preoperative DJ stent/nephrostomy (Table 1).

When patients were evaluated in terms of stone characteristics, there were no significant differences between the groups regarding stone laterality, pole location, number of stones, minimum/mean/maximum Hounsfield unit (HU) values, or stone radiopacity. Although the mean stone size was relatively larger in the FU group (11.9 ± 3.6 mm) compared with the FF group (11.2 ± 3.7 mm), the difference was not statistically significant (*p* = 0.054) (Table 1).

The operative time was 34.7 ± 8.7 min in the FF group and 42 ± 12.9 min in the FU group, being significantly shorter in the FF group (*p* < 0.001). No fluoroscopy was used in the FF group, whereas in the FU group the fluoroscopy time and dose were recorded as 7.75 ± 3.6 s and 1.31 ± 0.61 mGy, respectively. There were no significant differences between the groups in terms of length of hospital stay or SFR. No intraoperative complications were observed in either group. Postoperative complications occurred in 29 (21.9%) patients in the FF group and 42 (34.1%) patients in the FU group; the difference between groups was statistically significant (*p* = 0.030). Infectious complications, including fever, urinary tract infection (UTI), and sepsis, were significantly more frequent in the FU group (Table 2).

## 4. Discussion

Urolithiasis is a common health problem, affecting approximately 7% of the female population and 13% of the male population. Accounting for a substantial proportion of emergency department visits, urolithiasis also imposes a considerable burden on healthcare systems and the economy [13]. In the management of urolithiasis, minimally invasive procedures are rapidly replacing open surgery and have become the most effective treatment option even for larger stones in anatomically challenging systems. RIRS is one of the minimally invasive modalities used for the treatment of renal stones. One drawback of the RIRS procedure is that it is conventionally performed under fluoroscopic guidance. The use of fluoroscopy during RIRS plays a critical role in ensuring procedural safety [14]. Despite its benefits, during fluoroscopy-guided procedures both the surgical team and patients are exposed to radiation-associated risks, including tissue reactions as well as carcinogenic and hereditary effects. The radiation dose delivered to the patient during a fluoroscopy-guided procedure depends on the total fluoroscopy time; exposure parameters (tube voltage, tube current, and beam filtration); fluoroscopy mode; X-ray geometry; and the number of radiographic images acquired. In a multicenter study conducted by the Southeastern European Urolithiasis Research Group, patient radiation exposure during endoscopic urinary stone treatment ranged from 0.6 to 15.9 mGy [15]. In our study, the mean fluoroscopy dose in the FU group was calculated as 1.31 ± 0.61 mGy, which is consistent with the available literature. In all FF protocols, as patient safety is the primary concern, it is recommended that the C-arm be positioned so that it is readily available for use whenever necessary [16]. In our study, the C-arm was kept in a ready-to-use position within the operating room throughout the procedures. In our study, there was no requirement for emergency fluoroscopy in the FF group.

In recent years, as RIRS has been increasingly adopted for the treatment of urinary stone disease, the risk of radiation exposure for both patients and urologists have also risen, emerging as an important clinical concern [15,17]. Moreover, patients followed for urinary stone disease may require repeated CT scans and recurrent stone-related treatments such as extracorporeal shock wave lithotripsy, RIRS, and percutaneous nephrolithotomy [18,19]. For these reasons, efforts to reduce radiation exposure for both patients and urologists are warranted. In line with these efforts, fluoroscopy-free (FF) RIRS has been implemented for the treatment of uncomplicated renal stones, supported by technological advancements in endoscopic equipment. In 2015, Olgin et al. successfully performed 50 FF ureteroscopy procedures and concluded that FF ureteroscopic management can be effectively applied for upper urinary tract stones [11]. In retrospective studies, the risk of selection bias is high because patient allocation is not truly randomized. In our study, patients were allocated into two groups in a quasi-random manner based on their surgical appointment schedules. Peng et al. retrospectively evaluated the FF RIRS procedure in 140 consecutive patients with renal stones operated on by a single surgeon; the stone-free rate (SFR) at postoperative 1 month was 95.7%, and no major intraoperative complications were observed [20]. Alma et al., in their retrospective study including 190 patients, reported stone-free rates (SFR) at postoperative month 1 of 92.2% and 90.8% in the fluoroscopy-use (FU) and fluoroscopy-free (FF) groups, respectively (*p* = 0.724). No statistically significant differences were observed between the groups in terms of stone size (*p* = 0.752) or operative time (*p* = 0.108) [21]. Güner and Günaydın prospectively compared fluoroscopy-free (FF) RIRS (*n* = 67) and fluoroscopy-use (FU) RIRS (*n* = 58) in patients with renal stones <20 mm. They found no statistically significant differences between the two groups in terms of length of hospital stay, operative time, SFR, complication rate, analgesic use, need for additional treatment, or visual analogue scale score [22]. In our study, the stone-free rates (SFR) at postoperative month 1 were comparable between the groups, with 112 patients (84.8%) in the FF group and 107 patients (87.0%) in the FU group (*p* = 0.623), and our findings were consistent with the existing literature. There were no significant differences between the groups regarding stone size, number, location, or other stone-related characteristics.

In a multicenter prospective study including 128 patients, Chung et al. assessed the stone-free rate (SFR) at postoperative month 1 using CT. The success rate was 78% in the fluoroscopy-free (FF) RIRS group and 80% in the fluoroscopy-use (FU) RIRS group, with no statistically significant difference between the two groups [23]. Among the limitations of the study were variations in the types of semi-rigid ureteroscopes, ureteral access sheaths, and flexible ureterorenoscopes used. In our study, all procedures were performed by an experienced urologist in endourology using a single standardized semi-rigid ureteroscope and a single type of flexible ureterorenoscope. An experienced urologist was defined as having >10 years of endourology experience, having performed >500 endourological cases, or performing >100 endourological cases annually. A systematic review observed that fluoroscopy-free (FF) ureteroscopy is generally performed by experienced, high-volume endourologists in carefully selected patients, and it emphasized that junior trainees should exercise caution when considering FF endourology [24]. In the systematic review, the complication rate was 5.9% (*n* = 71/1201) in the fluoroscopy-free (FF) group and 12.6% (*n* = 149/1186) in the fluoroscopy-use (FU) group. Complication rates were significantly higher in the FU group (*p* = 0.009). In our study, the complication rate was 21.9% (*n* = 29) in the FF group and 34.1% (*n* = 42) in the FU group, with a statistically significant difference between the groups (*p* = 0.030). The higher complication rate observed in the FU group compared with the FF group was consistent with the findings of the systematic review. The groups differed with respect to fever, urinary tract infection, and sepsis. As operative time increases, the likelihood of infectious complications also rises. Accordingly, the FU group, in which complications were more frequently observed, also had a longer operative time. In both groups, pain was the most common complication. Overall complication rates in our study were higher than those reported in the systematic review. This discrepancy may be attributable to the inclusion of minor adverse events in our study, such as transient postoperative fever, mild pain not requiring significant analgesic therapy, and self-limiting mild hematuria. The rate of infectious complications in our cohort—including fever, urinary tract infections, and sepsis—was 10.5% (*n* = 27/255), which is consistent with the 2.2–13% range reported in the literature [25,26,27].

In a study evaluating 122 patients undergoing RIRS, Tinoco et al. observed a significantly longer operative time in the FU group (median, 55.0 min vs. 40.0 min; *p* < 0.001). The operating surgeons also included trainees/residents [28]. In our study, the operative time was 34.7 ± 8.7 min in the FF group and 42 ± 12.9 min in the FU group, and it was significantly shorter in the FF group (*p* < 0.01). Because all procedures in our study were performed by an experienced endourologist, operative times were relatively shorter overall. We believe that this difference in operative times between the two groups may be attributable to intraoperative positioning of the fluoroscopy equipment, interruption of procedural maneuvers while reviewing fluoroscopic images, and the additional time required to interpret these images.

The limitations of our study include its retrospective design, although patient allocation between groups was randomized. The single-center design of the study limited the evaluation of different equipment and hardware (e.g., various URS brands and laser types). Furthermore, the borderline significance in age distribution resulting from randomization introduced a degree of heterogeneity between the groups. Subgroup analysis revealed that this discrepancy originated specifically from patients younger than 40 years of age (*p* = 0.040). While having all procedures performed by a single experienced surgeon provided procedural standardization, it precluded assessment of feasibility and reproducibility when managed by less experienced surgeons. Radiation exposure was reported as the dose rate (mGy/s) calculated from the fluoroscopy unit’s air kerma and fluoroscopy time; therefore, future studies should consider the use of dosimeters to more accurately quantify patient and staff exposure. Additionally, stone analysis could not be performed during the postoperative follow-up of the patients. Finally, the assessment of stone-free status was heterogeneous due to the lack of routine computed tomography for all patients, which may have led to variability in detecting residual fragments. Nevertheless, it should be kept in mind that CT-based follow-up, although more sensitive for identifying residual stones, increases radiation exposure. Addressing these limitations in future prospective studies would strengthen the evidence and improve the robustness of the findings.

## 5. Conclusions

In appropriately selected patients with renal stones < 2 cm, fluoroscopy-free RIRS performed by experienced surgeons can be applied effectively and safely, with shorter operative times and lower complication rates. The key principle for reducing radiation exposure without compromising procedural safety is not only adequate surgical experience but also avoiding reluctance to use fluoroscopy when necessary, while adhering to the ALARA principle. Future well-designed studies with larger sample sizes are needed to definitively determine whether fluoroscopy-free RIRS can become a routinely and safely applicable approach in daily urological practice.

## Figures and Tables

**Table 1:** Demographic and Clinical Characteristics of the Patients and Stone Characteristics.

	FF Group (*n* = 132)	FU Group (*n* = 123)	*p*
Age, (year) mean ± SD	52.7 (±14.2)	55.5 (±16.2)	0.046
<40	31.9 (±5.7)	27 (±7.5)	0.040
40–65	51.9 (±6.4)	53.3 (±7.3)	0.201
≥65	71.2 (±5.8)	72.7 (±5.9)	0.236
Sex, *n* (%)			
Male	82 (62.1%)	62 (50.4%)	0.059
Female	50 (37.9%)	61 (49.6%)	
BMI, (kg/m^2^) mean ± SD	27.2 (±4.4)	27.8 (±5.9)	0.757
Hypertension, *n* (%)	37 (28%)	56 (45.5%)	0.004
DM, *n* (%)	26 (19.7%)	35 (28.5)	0.101
ASA, *n* (%)			0.259
I	19 (14.4%)	16 (13%)	
II	93 (70.5%)	93 (75.6%)	
III	16 (12.1%)	14 (11.4%)	
IV	4 (3%)	0 (0%)	
Anticoagulant Use, *n* (%)	18 (13.6%)	23 (18.7%)	0.271
Prior Stone Surgery, *n* (%)			
ESWL	10 (7.6%)	11 (8.9%)	0.691
URS	43 (32.6%)	41 (33.3%)	0.898
RIRS	28 (21.2%)	16 (13%)	0.083
PNL	11 (8.3%)	16 (13%)	0.225
Preop. catheterization *n* (%)			
DJ Stent	65 (49.2%)	62 (50.4%)	0.853
Nephrostomy	3 (2.3%)	3 (2.4%)	0.930
Stone laterality, *n* (%)			0.289
Right	52 (39.4%)	52 (42.3%)	
Left	73 (55.3%)	59 (48%)	
Bilateral	7 (5.3%)	12 (9.8%)	
Number of stones, *n* (%)			0.304
1	83 (62.9%)	68 (55.3%)	
>1	49 (37.1%)	55 (44.7%)	
Stone size (mm), mean ± SD	11.2 (±3.7)	11.9 (±3.6)	0.054
Stone radiodensity, (HU) SD			0.259
Minimum	291.4 (±280.1)	365.8 (±329.6)	
Mean	717.9 (±314.2)	694.6 (±305.5)	
Maximum	1066.3 (±394.2)	1012.6 (±397)	
Radiopacity, *n* (%)			0.985
Radiolucent	32 (24.2%)	30 (24.4%)	
Semi-opaque	10 (7.6%)	10 (8.1%)	
Radiopaque	90 (68.2%)	83 (67.5%)	
Stone location, *n* (%)			
Renal Pelvis	45 (34.1%)	39 (31.7%)	0.686
Lower calyx	33 (25%)	29 (23.6%)	0.791
Middle calyx	17 (12.9%)	15 (12.2%)	0.869
Upper calyx	8 (6.1%)	7 (5.7%)	0.900
Multiple locations, *n* (%)	29 (21.9%)	33 (26.8%)	0.366

**Table 2:** Postoperative Outcomes of the Patients.

	FF Group (*n* = 132)	FU Group (*n* = 123)	*p*
Operative time, (min) mean ± SD	34.7 (±8.7)	42 (±12.9)	<0.001
Fluoroscopy time, (s) mean ± SD	0 (0)	7.75 (±3.6)	<0.001
Fluoroscopy dose, (mGy) mean ± SD	0 (0)	1.31 (±0.61)	<0.001
Hospital Stay, (day) mean ± SD	1.7 (±2.3)	1.8 (±2.4)	0.993
SFR, *n* (%)	112 (84.8%)	107 (87%)	0.623
Complications, *n* (%)	29 (21.9%)	42 (34.1%)	0.030
Pain	13 (9.8%)	15 (12.1%)	0.549
Fever	3 (2.3%)	7 (5.7%)	0.160
Hematuria	7 (5.3%)	9 (7.3%)	0.508
UTI	6 (4.5%)	10 (8.1%)	0.238
Sepsis	0 (0%)	1 (0.8%)	0.299

## Data Availability

The data presented in this study are available on request from the corresponding author. The data are not publicly available due to patient privacy and ethical restrictions.

## Abbreviations

The following abbreviations are used in this manuscript:

RIRS	Retrograde Intrarenal Surgery
FF	Fluoroscopy-free
FU	Fluoroscopy-used
SFR	Stone-free rate
ALARA	As low as reasonably achievable
UTI	Urinary tract infection
